# CNS Toxicity of Immunotherapy

**DOI:** 10.16966/2469-6714.157

**Published:** 2020-06-24

**Authors:** David Gorlin, Rania Bilwani, Saman Khan, Maryam Gul, Muhammad H Imam, Ammar Chaudhry

**Affiliations:** 1 Research Intern, Department of Diagnostic Radiology, City of Hope National Cancer Center, Los Angeles, California, USA; 2 Amaze Research Foundation, Los Angeles, California, USA; 3 Florida Cancer Specialists, Orlando, Florida, USA; 4 Director, Precision Imaging Lab, Director of Imaging Informatics Research, Department of Diagnostic Radiology City of Hope National Cancer Center, Los Angeles, California, USA

**Keywords:** Immunotherapy, Imaging biomarkers, CNS toxicity

## Introduction

Recent advancements in cancer treatment have significantly contributed to the improvement of the cancer mortality rate and the overall relative survival rate [1]. Immunotherapy has emerged as a promising treatment option alongside surgery, chemotherapy, and radiotherapy. Immunotherapy is a unique treatment strategy that works by engaging the immune system to elicit anti-tumor response. In recent years, Immune Checkpoint Inhibitors (ICI) agents alone or in combination with other treatment options have demonstrated the capacity to eliminate cancers with greater efficacy and tolerability than previous standard-of-care therapies [2]. Thus, ICI and cell-based immunotherapies have revolutionized the treatment of a range of hematological and solid cancer types (Table 1).

Despite novel insight into the mechanics of successful immunotherapy, a number of obstacles hinder optimal clinical performance, including a broad variety of Immune-Mediated Adverse Events (IMAE). To maintain durability against tumors, immunomodulators must elicit a powerful immune response. Consequently, the heightened immune activity may lead to inflammation, triggering an autoimmune response which results in damage to healthy cells and induces adverse events across the body [19,20]. The frequency and severity of IMAEs vary by site and type of immunotherapy. According to the National Cancer Institute of the National Institutes of Health’s Common Terminology Criteria for Adverse Events (version 5.0) [21], the severity of immune-mediated adverse events are classified as: Table 2.

IMAEs in the Central Nervous System (CNS) present a significant obstacle for the continuation of treatment and favorable patient outcomes. Immunotherapies can impair the regulatory mechanisms in the CNS that protect it from excessive immune responses, which may cause severe neuronal damage. These toxicities can escalate rapidly and without proper management can be fatal. The incidence of IMAEs in the CNS varies by immunotherapy type, with highest reported rates in CAR T-cell therapy (0–87%) [22] and ICI therapy (3.8–12%) [23]. The pathogenesis of immune-mediated CNS toxicities is an active area of research and has been associated with: blood-brain barrier disturbance, excessive cytokine concentration in the blood and cerebrospinal fluid, and endothelial activity [22,24].

Improved understanding of the pathogenesis of CNS toxicities may reveal biomarkers capable of assessing risk and severity, as well as distinguishing CNS toxicity from tumor-related damage. In addition, predictive biomarkers could lead to earlier detection and prevent treatment-mediated toxicities. While model biomarkers have been explored (e.g. IL6 and MCP-1 [24], lymphodepletion [25], IL15, IL10, and EFG [26]), their significance and reliability require further validation. Noninvasive biomarkers have many advantages over traditional invasive techniques, and can serve as a guide for clinicians to describe the nature of immune-mediated CNS toxicity and track its progress.

IMAEs in the CNS can be noninvasively diagnosed and monitored using conventional ^18^F-fluorodeoxyglucose (FDG), ^18^Fluoro-3’deoxythymidine (FLT), or ^18^Fluoro-ethyl-L-tyrosine (FET) Positron Emission Tomography/Computed Tomography (PET/CT); contrast enhanced CT, and Magnetic Resonance Imaging (MRI). FDG PET/CT is the most commonly utilized modality to analyze IMAEs. Because FDG radiotracer is a glucose analogue, uptake values will increase in both inflammatory cells and tumor; allowing it to detect and localize IMAEs in the CNS [27]. MRI is the favored imaging modality for IMAEs in the CNS because of higher spatial resolution and soft tissue resolution, relative to PET/CT [28,29]. Radiological patterns of IMAEs may overlap with other pathologies, and it is essential to differentiate IMAEs from tumor-related damage or other potential conditions caused by infections, metabolism, or neoplastic and paraneoplastic complications [30].

Majority of IMAEs in the CNS develop within the first few months of immunotherapy; however, they can present late or possibly after discontinuation [20,23]. Recognition and management are crucial to prevent or de-escalate any IMAEs. For grade 2 and above symptoms, treatment should be withheld. Grade 3 and above symptoms generally require discontinuation of treatment [28]. Immunosuppressive corticosteroid drugs are the recommended treatment for IMAEs; however, when administered simultaneously with immunotherapy, corticosteroids reduce its efficacy [28]. The reversibility of IMAEs are determined by their pathogenesis. Even after discontinuation of treatment, IMAEs may persist [20]. Reversibility patterns displayed on imaging modalities can help improve patient management. According to the Immunotherapy Response Assessment in Neuro-Oncology guidelines, clinicians may consider resuming immunotherapy treatment given that follow-up images confirm safety and patients show positive results with acceptable toxicity [28].

In this review, we summarize the immune-mediated CNS toxicities, with primary focus on their clinical symptoms, biomarkers, and radiological appearance. By examining the IMAEs in the CNS according to the type of immunotherapy, we hope to assist clinicians in earlier recognition and improved patient management.

## Immune Checkpoint Inhibitors

The use of ICIs for immunotherapy has revolutionized cancer treatment because of its efficacy in improving prognosis. Ipilimumab is an ICI that targets cytotoxic T lymphocyte-associated antigen 4 (CTLA-4), which limits T cell activation by competing with the costimulatory protein CD28 for binding [31]. Other ICIs have different targets, such as programmed cell death receptor (PD-1) or its ligand (PD-L1) (Table 1). T cell immunoglobulin and mucin domain-containing protein 3 (TIM-3), lymphocyte-activation gene 3 (LAG-3) and V domain Ig suppressor of T cell activation (VISTA) are still being studied as targets [32]. IMAEs associated with the use of ICIs have since been discovered and are being investigated.

Although Neurological Adverse Events (NAEs) are rare, they are still a relevant area of research because of the significant possibility of morbidity and mortality. Various CNS toxicities are associated with ICIs (Table 3), most commonly with ipilimumab [31]. Toxicity should be managed by interrupting the use of ICIs and using corticosteroid immunosuppression as needed [37]. This should be followed by other T cell suppressors and intravenous immunoglobulin (IVIG) or plasmapheresis for severe toxicities (grade 3 and higher) [38]. Imaging plays a critical role in identifying toxicity; early diagnosis and intervention of Paraneoplastic Syndromes (PNSs) can help prevent irreversible effects [32]. Diagnostic specificity is essential in differentiating between aseptic meningitis, which can show leptomeningeal enhancement on MRI, and leptomeningeal disease, which has sulcal enhancement on T1 contrast-enhanced and FLAIR sulcal hyperintensity [31].

Novel ICI-based combinatorial approaches in cancer treatment have presented the best possible patient outcomes for a number of cancers to date. By combining ICI with standard treatments and other immunotherapies, cancer cells are eliminated via cytotoxic and cytostatic mechanisms as well as the enhanced immune response; increasing response rates to over 50% [39]. The considerable potential of synergistic combinatorial approaches is exemplified by outcomes in patients with Non-Small Cell Lung Cancer (NSCLC). When administered anti-PD1 antibodies in addition to chemotherapy, the overall response rate of patients with NSCLC rose from 19% to 48% with no significant increase in adverse events (ClinicalTrials.gov Identifier: NCT02578680). ICIs demonstrate the ability to optimize the efficacy of standard treatment options, including chemotherapy, radiotherapy, and radiotherapy.

Blocking CTLA-4, PD1 and PD-L1 antibodies allows the immune system to bypass several tumor evasion mechanisms simultaneously, resulting in increased infiltration, activation and cytokine production [39]. This treatment strategy overcomes immunosuppressive mechanisms in the tumor microenvironment and augments cancer cell elimination. However, as a consequence of compounded autoimmunity, the occurrence of immune-mediated AEs (Table 3) increases to approximately 12% [30].

## Car T Cells

Chimeric Antigen Receptor (CAR) T cell therapy is a type of gene therapy designed to recruit the body’s own immune system to respond to proliferating tumor cells. This is accomplished through ex vivo augmentation of antigen receptors found on autologous T cells, which are cultured and transplanted back into the patient [40]. Since FDA approval in 2017, commercially available CAR T-Cells have significantly improved the management of hematological malignancies and have garnered CMS national determination coverage as of September 2019. Efforts to adapt this treatment to a myriad of other tumor types is currently being investigated.

The first FDA approved commercial CAR T-Cell therapy used genetically modified chimeric antigen receptors that target CD-19 positive malignant B-Cells to treat refractory acute lymphoblastic leukemia [41,42]. Investigators have since demonstrated its variegated therapeutic applications in the treatment of other CD 19 positive malignancies such as Diffuse Large B Cell Lymphoma [43–45]. CD-19 was chosen as a target for therapy due to its expression early in the B cell lineage [40], and while anti-CD-19 CAR T-cell therapy has demonstrated a high rate of remission, BCMA and antiCD22 CAR T-cells have also been developed as a next line therapy in situations where relapse occurs. This has been of particular benefit in cases of pediatric B-ALL [12,46] and in Multiple Myeloma [14].

With positive results in hematologic malignancies, there is increasing interest in implementing CAR T-Cell therapy towards treating solid tumors as well. Clinical trials demonstrating the benefit IL13Rα2 CAR T-cells in the treatment of glioblastoma are currently underway [13]. Furthermore, HER-2 specific CAR T-Cells have been successfully designed to target breast cancer metastatic to the brain [47], and phase 1 clinical trials have begun to investigate their safety and efficacy in patient care (ClinicalTrials.gov Identifier: NCT03696030).

In addition to CAR T-Cell therapies, Bispecific T Cell engagers (BITE) such as blinatumomab have been developed to recruit T cells in a similar fashion without the demanding workflow of gene therapy. Blinatumomab is a monoclonal antibody that shares affinity for both CD19 on malignant B cells and CD3 on cytotoxic T-cells generating a similar mechanism of action as antiCD 19 CAR T-cells. Bispecific T-Cell engagers are often considered alongside CAR T-cells given their overlap in treatment indication, mechanism of action, and toxicity management [48].

Even in their nascent clinical trials, each of these breakthrough immunotherapies has exhibited similar toxicities (Table 4) that complicated patient care [41,43,45,49]. Within two to six days of receiving treatment, patients would report a cluster of symptoms that have become known as cytokine release syndrome, or CRS [50–53].

The pathogenesis of CRS is best described by the duly named cytokine storm that results from the rapid activation of the immune system flooding the blood circulation with TNF alpha, IL1, IL6, IL8, IL 10, and interferon gamma [52,54,55]. Uncontrolled, severe CRS can result in hypotension, organ failure, and death (Table 4). However, approval to use anti IL6 tocilizumab for CRS has lowered its incidence and severity [51]. Glucocorticoids are also used as a second line when tocilizumab fails. There are ongoing studies investigating the timing of tocilizumab and its efficacy in treating CRS specifically in B cell ALL (ClinicalTrials.gov number, NCT02906371); however, neither glucocorticoids nor anti IL6 therapy have been reported to significantly effect treatment response.

The second most common complication of Cellular Therapy is neurotoxicity, and is experienced by 40–60% of patients [48]. Headaches caused by encephalopathy were commonly reported and believed to be included in the presentation of CRS, becoming known as cytokine release encephalopathy-syndrome [27,56]. However, further analysis revealed several patients have experienced varying degrees of neurotoxicity, sometimes in the absence of CRS, and so these discrete symptoms have become known as Immune Effector Cell-Associated Neurotoxicities (ICANs; Table 5) [48,51]. Furthermore, in the phase II trial investigating the use of Axicabtagene ciloleucel in large B-Cell Lymphoma, specific biomarkers such as IL-2, GM-CSF, and ferritin were exclusively detected in the presence of grade 3 or greater neurological events [43]. This argued for classifying ICANs independently of CRS.

There have been several different grading systems developed specifically for neurological toxicity, which unfortunately have led to inconsistencies throughout the literature [51]. The most accepted diagnostic grading system today is the consensus grading scale outlined by the American Society for Transplant and Cellular Therapy [53] (Table 5). This incorporates either a revised version of the CARTOX-10 neurological exam now known as Immune Effector Cell Encephalopathy (ICE) (Table 6) grading tool, or the Cornell Assessment of Pediatric Delirium (CAPD) for children under the age of 12. Grading also considers seizure evaluation, as well as biomarkers to determine the severity of ICANS. Grade 5 neurotoxicity by convention is considered to result in death.

Neurotoxicity is an on-target effect of CAR-T cell therapy, and reported in all varieties of treatment [40]. CAR-T cell expansion in the peripheral blood is a strong prognostic indicator of treatment response [43,45]. Independently, both the peak of CAR T-Cell expansion [43] and severity of CRS [26] are significantly associated with the onset of grade 3 or higher neurologic events. Neurotoxicity by itself is managed empirically through supportive care. In long term follow up of patients with ICANs, neurotoxicity is not correlated with CNS progression of disease and irreversible damage is very rarely observed in patients who have recovered [48,51].

Radiographically, MRI of the brain (Table 7) tends to be normal during low grade neurological events, although patterns of vasogenic edema, multifocal microhemorrhage, and leptomeningeal enhancement have been demonstrated in those who have experienced moderate-to-severe neurotoxicity ≥ grade 3 [25,26,57]. Overall, these patterns have been found to be similar to those characteristic of posterior reversible encephalopathy syndrome (PRES) [26,57]. In some cases, local and diffuse cerebral Radiographically, MRI of the brain (Table 7) tends to be normal during low grade neurological events, although patterns of vasogenic edema, multifocal microhemorrhage, and leptomeningeal enhancement have been demonstrated in those who have experienced moderate-to-severe neurotoxicity ≥ grade 3 [25,26,57]. Overall, these patterns have been found to be similar to those characteristic of posterior reversible encephalopathy syndrome (PRES) [26,57]. In some cases, local and diffuse cerebral edema have been identified that result in cortical damage or death in the most severe cases of neurotoxicity. There have also been reports of transient exacerbation of previously identified lesions present at the start of treatment, which include damage incurred during chemotherapy or radiation. There currently is a lack of high-powered controlled studies investigating these neuroimaging patterns. This has warranted the call by many to further investigate the role of neuroimaging in the standard of care for patients undergoing cellular therapies [58–60].

There remains to be an ongoing discussion regarding the pathogenesis of ICANS. Analysis of samples of CSF biopsied through lumbar puncture during high grade neurologic events have revealed elevated protein levels, consistent with disruption of the blood brain barrier [25,26]. Further analysis of biomarkers in the CSF also revealed abnormally high ratios of inflammatory cytokines such as IL6 compared to peripheral circulation, suggesting an increased de novo production within the CNS. NDMA agonists have also been hypothesized to contribute to an increase in blood brain permeability and neurological symptoms [26].

Many of the toxicities reviewed have been reported in the use of anti CD19 CAR T-Cells, as these are the most widely used in current practice. There has been some debate as to whether variability in the costimulatory domain of anti CD19 CAR T-Cells could increase neurotoxicity. Originally, higher incidence of fatal cerebral edema occurred in anti CD19 CAR T-Cells containing CD28 co-stimulatory domains than in those containing 41BB domains [42,43]. However, there is insufficient evidence to suggest one is more toxic than the other. ICANs have been reported to be common among all types of CAR T-Cell therapy, although the incidence of severe high-grade neurological events is less common in those other than anti CD-19. For example, cerebral edema has been reported in BCMA therapy, but this appears to be more rare in antiCD-22 CAR T-Cell therapy [40]. In the treatment of solid tumors, grade 1–2 neurotoxicity was reported in treatment of glioblastoma with IL13Rα2 CAR T-cells [13]. An appreciable increase in inflammatory cytokine concentration in the CSF was consistent with other CAR T-cell therapies, although severe neurotoxicity has yet to be reported.

CAR T-cell and other immune effector cellular based therapies is a rapidly emerging approach to treating cancer, with neurotoxicity being one of its most common side effects. Most common signs of neurotoxicity are headaches and/or disorientation. The role biomarkers such as CSF analysis and neuroimaging patterns can play an important role in the management of this neurotoxicity. Moving forward, standardized toxicity grading and diagnostic protocols will be impetrative in understanding neurotoxicity pathogenesis, prevention, and management.

## Stem Cell Therapy

Hematopoietic Stem Cell Transplants (HSCT) have contributed greatly to the treatment of cancer, but the pre-conditioning required consists of chemotherapy and/or radiotherapy, which can lead to various CNS toxicities such as severe encephalopathy, posterior reversible encephalopathy syndrome (PRES), and transverse myelitis [61]. HSCT has pioneered concepts of stem cell therapy as cancer treatment [62].

Induced Pluripotent Stem Cells (iPSCs) prevent adverse drug reactions by personalizing treatment specific to the patient. iPSCs can be used to generate anti-tumor T and Natural Killer (NK) lymphocytes. iPSCs are particularly advantageous due to their ability to be genetically modified, making them useful to a wider range of patients and cancers [63]. Because stem cells are unspecialized, they can theoretically differentiate into any cell type. Using this characteristic of iPSCs, scientists can noninvasively develop models of otherwise inaccessible tissues and cell types of organ systems such as the nervous system [64]. These organoids can be derived from iPSCs and serve as advanced, patient-specific models to help create personalized treatment by allowing researchers to determine the efficacy and toxicity of a particular drug [65].

The tumor-tropic nature of human Neural Stem Cells (NSCs) allows for direct therapeutic targeting of brain tumors without producing significant toxicity of normal brain tissue. A study by Portnow J, et al. found no dose-limiting toxicity associated with the intracranial administration of cytosine deaminase neural stem cells (CD-NSCs) into recurrent high grade glioma patients, proving NSCs to be a safe and effective brain tumor therapy [66].

## Dendritic Cell Therapy and Oncolytic Viral Therapy

Dendritic Cell (DC) Vaccines have shown promise to aid in treating cancer. Not only are they effective, but they show minimal toxicity compared with other forms of immunotherapy. Dendritic cells are a unique type of immune cell that have the capacity to regulate both innate and adaptive immunity. DCs work by stimulating the body’s immune system to kill cancer by recognizing cancer cell antigens and activating T-cells to eradicate the tumor. Under normal circumstances, DCs remain inactive until they are exposed to a stimulus such as inflammatory cytokines, microbial factors or endogenous alarmins. Once DCs are activated, they produce antigens to stimulate T cells on their major histocompatibility molecules. DC vaccines working against tumor antigens work to create an arm that aims to boost one’s immune response through the use of immunotherapy. Cell-based therapies are emerging as a possible cancer cure because they have fairly low levels of toxicity as well as their ability to activate other immune modulators such as Natural Killer cells and T cells [67]. A study conducted at Rockefeller University, using ex vivo-generated monocyte-derived DCs, showed that neuropathy was one of the major toxicities associated with dendritic cell vaccines (ClinicalTrials.gov Identifier: NCT00345293).

Oncolytic viral therapy is another promising aid in cancer treatment. This immunotherapy has the advantage of the virus being able to infect and replicate in the tumor cells [68]. Neurotoxicities associated with this therapy include; dizziness, headache, Neurological disorder NOS, neuropathy, speech disorder, and syncope vasovagal. Patients also experienced vascular toxicities that consisted of flushing, hemorrhage, hypotension, and thrombosis (ClinicalTrials. gov Identifier: NCT00408590). Causing similar blood brain barrier disruption very novel approaches in curing cancer. These mechanisms overlap with some other treatments but some are nonspecific such as neuropathy. While some of these treatments overlap with ones previously mentioned, the appearance of neuropathy raises some questions about the on target effects of oncolytic viral therapy.

## Conclusion

While immunotherapy presents the opportunity to eliminate cancers with great efficacy, the non-specific, hyperactive immune responses may target self-antigens and inflammatory cytokines. IMAEs in the CNS can cause serious cognitive and physiological impairments, posing limitations that undermine immunotherapy’s success in clinical trials. Taking adverse events into consideration, patients should be closely monitored for clinical symptoms and suspicious radiographic abnormalities. PET/CT, CT, and MRI are readily available imaging modalities that can noninvasively identify IMAEs and display biomarkers, which may allow clinicians to describe their grade and reversibility. Early case series have shown positive MRI findings in patients with moderate-to-severe CNS toxicities. As such, clinicians can use imaging as a guide to make informed decisions regarding patient management, especially in critically ill patients. Depending on grade and reversibility of the IMAEs, clinicians may need to interrupt the immunotherapy dose schedule and/or administer corticosteroids to prevent the effects of the adverse events from outweighing the benefits derived from the treatment. Until progress is made in identifying the safest targets for antigen-specific cancer immunotherapies, early recognition and management of IMAEs in the CNS can prevent patient suffering and improve patients’ quality of life.

## Figures and Tables

**Table 1:** FDA approved indications of immunotherapy.

Type of Immunotherapy	FDA approved indications
**Immune Checkpoint Inhibitors**	
Anti PD-1	
Pembrolizumab [3]	Non-Small Cell Lung Cancer, Head and Neck Squamous Cell Carcinoma, Melanoma
Nivolumab [4]	Small Cell Lung Cancer, Advanced Renal Cell Carcinoma, Classical Hodgkin Lymphoma
Cemiplimab [5]	Cutaneous Squamous Cell Carcinoma
Anti PD-L1	
Atezolizumab [6]	Non-Small Cell Lung Cancer, Advanced Renal Cell Carcinoma, Triple-Negative Breast Cancer
Avelumab [7]	Advanced Renal Cell Carcinoma, Urothelial Carcinoma, Merkel Cell Carcinoma
Durvalumab [8]	Non-Small Cell Lung Cancer, Urothelial Carcinoma
Anti CTLA-4	
Ipilimumab [9]	Melanoma
Combination of Ipilimumab and Nivolumab [9]	Hepatocellular carcinoma, Advanced renal cell carcinoma, Melanoma, Microsatellite instability-high (MSI-H) or mismatch repair deficient (dMMR) metastatic colorectal cancer
**CAR T-cell Therapy**	
Anti-CD19	
Tisagenlecleucel [10]	B-cell acute lymphoblastic leukemia, Diffuse large B-cell lymphoma, High grade B-cell lymphoma, DLBCL arising from follicular lymphoma
Axicabtageneciloleucel [11]	Diffuse large B-cell lymphoma, Primary mediastinal large B-cell lymphoma, High grade B-cell lymphoma, DLBCL arising from follicular lymphoma
Anti-CD22 [12]	Currently under investigation for B-cell acute lymphoblastic leukemia
IL13Rα2 [13]	Currently under investigation for Glioblastoma
Anti-BCMA [14]	Currently under investigation for relapsed or refractory multiple myeloma
Anti-CD123 [15]	Currently under investigation for acute myeloid leukemia
**Vaccines**	
Sipuleucel-T [16]	Metastatic castrate resistant (hormone refractory) prostate cancer
BCG Live [17]	Carcinoma in situ (CIS) of the urinary bladder, papillary tumors
**NK cell**	
CAR-NK-92 [18]	Currently under investigation for acute myeloid leukemia, glioblastoma, prostate cancer, and ovarian cancer

**Table 2:** Common terminology criteria for adverse events (version 5.0) [21].

Grade	Classifications	Severity
1	Asymptomatic or mild symptoms Clinical or diagnostic observations only No intervention required	Mild
2	Minimal, local or noninvasive intervention required Limits activities of daily life	Moderate
3	Hospitalization required Disabling or limiting self care activities of daily life	Severe/medically significant but not immediately life-threatening
4	Urgent intervention required	Severe, life-threatening
5	Death related to adverse events	Extremely severe, life-threatening

**Table 3:** Immune-mediated CNS toxicities associated with immune checkpoint inhibitors.

Toxicity	Toxicity grade	Imaging Features	Symptoms
Hypophysitis	3–4 [33]	MRI: Enlargement of pituitary gland/stalk and hypoenhancing nodules; heterogeneous enhancement [31]	Headaches, dizziness, diplopia, loss of peripheral vision and fatigue, nausea/vomiting [31] hyponatremia (diabetes insipidus) or multiple endocrinopathy
Encephalopathy/encephalitis	3–4 [34]	MRI: normal in ½ patients; medial temporal T2 hyperintensity, negative CSF [32] enhancement of gray matter or cortex; white matter t2 hyperintensities	Confusion, decreased arousal, language deficit, seizures, gait instability, headaches, fevers, and hallucinations [32]
Aseptic meningitis	3–4 [34]	MRI: Typically normal imaging but you may see leptomeningeal enhancement, CSF analysis may show lymphocytic pleocytosis [32]	Worsening headache, photophobia, neck stiffness, possible fever, vomiting but normal CNS function [32]
CNS demyelination	3–4 [35]	T2 hyperintensity of white matter tracts +/− enhancement (multiple sclerosis type pattern) [32]	Confusion [32]
Paraneoplastic syndrome	3–4 [34]	MRI: Normal or T2 hyperintensity in bilateral medial temporal lobe [32]	Various neurological symptoms including slurred speech and vision problems [36]
Transverse myelitis		Focal T2 abnormality and CSF lymphocytosis [32] Spine MRI T2 intense lesion, T1C signal abnormality [31]	Motor, sensory, and/or autonomic dysfunction [31]

**Table 4:** ASBMT CRS consensus grading.

Grade	CRS Parameter
1	Fever: Temperature ≥ 38°C
2	Fever: Temperature ≥ 38°C Hypotension: Does not require vasopressors And / or hypoxia: Requires low-flow oxygen delivery
3	Fever: Temperature ≥ 38°C Hypotension: Requires single vasopressor therapy (vasopressin) And / or hypoxia: Requires high-flow oxygen delivery
4	Fever: Temperature ≥ 38°C Hypotension: Multiple vasopressor therapy without vasopressin And / or hypoxia: Requires positive pressure oxygen therapy

**Table 5:** ASBMT ICANS consensus for adults.

Grade	Motor Findings	Elevated ICP/ cerebral Edema	Seizure Severity	Arousal level	ICE score age ≥ 12 years	CAPD score age < 12 years
1	None	None	None	Spontaneously awakens	7–9	1–8
2	None	None	None	Aroused by voice	3–6	1–8
3	None	Focal/ local edema on MRI	Any nonlife -threatening clinical seizure or nonconvulsive seizure on EEG	Arousal requires somatosensory stimulation	0–2	≥ 9
4	Focal impairment of voluntary movement (e.g. hemiparesis or paraparesis)	Diffuse cerebral edema on MRI; decerebrate or decorticate posturing; or cranial nerve VI palsy; or papilledema; or Cushing’s triad	Prolonged life-threatening seizure lasting more than 5 min; ore repetitive seizures without return to baseline	Unarousable, or requires intense physical stimulation. Coma	Unresponsive	Unresponsive

**Table 6:** ASBMT ICANS consensus grading: encephalopathy assessment tools.

CAPD: for children age < 12 years	ICE: age ≥ 12 years	
Rate each of the following from 0–4, 0 being always 4 being never		Orientation: Can recall the month, year, hospital, or city 4 points
Eye Contact	Restlessness	Naming: Demonstrates the ability to name up to 3 objects in the room objects 3 points
Purposeful actions	Inconsolable	Follows Directions: Performs a simple task on command: 1 point
Awareness of surroundings	Activate while awake	Writing: Demonstrates ability to write a complete sentence: 1 point
Communicates wants and needs	Extended response time	Attention: Counting backwards by 10 from 100: 1 point

**Table 7:** ICANs Associated with CAR T-cell Therapy.

Neurotoxicity	Grade	Neuroimaging Pattern	Source
Limbic encephalitis	3–4	MRI: T2 Flair changes in bilateral mesial temporal lobes, but also occasionally found in frontal and parietal lobes, and cerebellum	[59]
Cytotoxic edema	3–4	MRI: Cortical diffusion restriction on DWI and cortical swelling on FLAIR	[25,57]
Cortical injury	3–4	MRI: One patient with patient with cytotoxic edema reportedly progressed to have T1 hyperintensities in the cortical ribbon 10 days later. Another pediatric patient with diffusion restriction in the right occipital demonstrated occipital hypometabolism on FDG PET 10 months following acute injury	[25,57]
Vasogenic edema	3–5	MRI: FLAIR hyperintensity in bilateral thalami and brainstem (midbrain, pons, medulla, basal ganglia, extreme capsule, brachium pontis). No diffusion restriction. Punctate hemorrhage T2 dark lesions	[25,26,57]
Transient lesions of the splenium of the corpus collosum	3–5	MRI: Restricted diffusion on DWI and T2/ FLAIR hyperintensity	[26,59]
Global edema	5	MRI: Blurring of gray-white junctions and slit-like ventricles on FLAIR imaging	[25]
Aseptic meningitis	5	MRI: Diffuse leptomeningeal enhancement T2 hyperintensities	[25]
